# A pilot-scale microwave technology for sludge sanitization and drying

**DOI:** 10.1016/j.scitotenv.2017.06.004

**Published:** 2017-12-01

**Authors:** Peter M. Mawioo, Hector A. Garcia, Christine M. Hooijmans, Konstantina Velkushanova, Marjana Simonič, Ivan Mijatović, Damir Brdjanovic

**Affiliations:** a Department of Environmental Engineering and Water Technology, UNESCO-IHE Institute for Water Education, Westvest 7, 2611 AX Delft, The Netherlands; b Pollution Research Group, University of KwaZulu-Natal, Durban, 404, South Africa; c Faculty of Chemistry and Chemical Engineering, University of Maribor, Smetanova 17, 2000 Maribor, Slovenia; d Tehnobiro d.o.o., Heroja Nandeta 37, 2000 Maribor, Slovenia; e Department of Biotechnology, Delft University of Technology, Julianalaan 67, 2628 BC Delft, The Netherlands

**Keywords:** Waste activated sludge, Septic tank sludge, Faecal sludge, Microwave treatment, Pathogen reduction, Volume reduction

## Abstract

Large volumes of sludge are produced from onsite sanitation systems in densely populated areas (e.g. slums and emergency settlements) and wastewater treatment facilities that contain high amounts of pathogens. There is a need for technological options which can effectively treat the rapidly accumulating sludge under these conditions. This study explored a pilot-scale microwave (MW) based reactor as a possible alternative for rapid sludge treatment. The reactor performance was examined by conducting a series of batch tests using centrifuged waste activated sludge (C-WAS), non-centrifuged waste activated sludge (WAS), faecal sludge (FS), and septic tank sludge (SS). Four kilograms of each sludge type were subjected to MW treatment at a power of 3.4 kW for various time durations ranging from 30 to 240 min. During the treatment the temperature change, bacteria inactivation (*E. coli*, coliforms, *Staphylococcus aureus*, and *enterococcus faecalis*) and sludge weight/volume reduction were measured. Calorific values (CV) of the dried sludge and the nutrient content (total nitrogen (TN) and total phosphorus (TP)) in both the dried sludge and the condensate were also determined. It was found that MW treatment was successful to achieve a complete bacterial inactivation and a sludge weight/volume reduction above 60%. Besides, the dried sludge and condensate had high energy (≥ 16 MJ/kg) and nutrient contents (solids; TN ≥ 28 mg/g TS and TP ≥ 15 mg/g TS; condensate TN ≥ 49 mg/L TS and TP ≥ 0.2 mg/L), having the potential to be used as biofuel, soil conditioner, fertilizer, etc. The MW reactor can be applied for the rapid treatment of sludge in areas such as slums and emergency settlements.

## Highlights

• Microwave-based technology is promising option for treatment of faecal sludge, septic sludge and waste activated sludge.
• It is rapid and efficient in sludge sanitization (pathogen reduction) and drying (volume reduction up to 96%).
• The process generates useful end-products (dry sludge with energetic value of up to 23 MJ/kg and sterile condensate).
• Microwave-based treatment technology shows great potential to be applied to situations with high sludge generation rate.

## Graphical abstract

**Figure:**
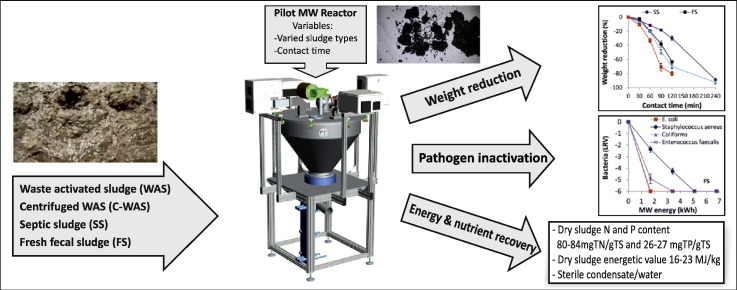

## 1 Introduction

Various waste materials (e.g. sludge) are generated from onsite sanitation systems and wastewater treatment facilities. Onsite sanitation technologies, particularly portable toilets, pit latrines, septic tanks, among others, are commonly applied in densely populated areas (e.g. slums and emergency settlements). Therefore, these systems are intensively used requiring frequent emptying (Brdjanovic et al., 2015), which results in large quantities of fresh faecal sludge (FS) and septic tank sludge (SS). Conventional wastewater treatment plants (WWTP) are also sources of high sludge quantities which are usually produced during primary and biological treatment (Hong et al., 2004). The large volumes of sludge from both the onsite sanitation systems (e.g. FS and SS) and the conventional WWTPs (e.g. waste activated sludge) with high number of pathogens, and high portion of water and organic matter in it can lead to excessive handling and disposal costs, disease outbreaks, and offensive odor (Mawioo et al., 2016a, Mawioo et al., 2016b). In addition, vector attractions are major concerns during the final disposal of the sludge (Hong et al., 2004).

These concerns are particularly occurring in areas with rapid sludge generation, requiring responsive collection, transport, and treatment. Rapid sludge generation limits the application of traditional alternatives for sludge treatment and/or disposal. For example, the existing FS treatment options, e.g. composting, co-composting with organic solid waste, conventional drying, anaerobic co-digestion with organic solid waste, and co-treatment in wastewater treatment plants (Ingallinella et al., 2002, Katukiza et al., 2012, Ronteltap et al., 2014) are mostly suited for regular sanitation contexts, and they have limitations to their application in highly populated areas (Mawioo et al., 2016a, Mawioo et al., 2016b). Of main concern is the relatively low conversion rate of the processes involved in these technologies which usually have difficulties to match the high sludge production rates in such areas. Furthermore, the common sewage sludge treatment and/or disposal options e.g. landfill, agricultural application, ocean dumping, etc. (Menéndez et al., 2002, Lin et al., 2012) are facing increasing pressure due to lack of land space for landfills and the stricter regulations regarding pollution of the farmlands and water bodies. Other alternatives applicable for sewage sludge such as anaerobic digestion, composting, etc., which have relatively low conversation rates of the processes involved may not be viable where rapid sludge processing is required. These limitations demonstrate the need for development of technologies that are adapted to the specific field conditions and that should address the priority issues in sludge management with the aim to substantially reduce pathogen and sludge volume and, if desired, sludge stabilization. However, the reduction of the number of pathogenic organisms is priority, particularly in the densely populated conditions, due to the eminent risk of disease outbreaks. Moreover, because the densely populated conditions, e.g. slums and emergency settlements are often characterized by constraints in land space and time, it is required that the applied treatment technology is rapid and efficient, compact, and easy to install and deploy.

A promising technology to achieve these requirements is the use of a MW based technology (Mawioo et al., 2016a). The MW energy is a part of the electromagnetic spectrum with wavelengths (λ) ranging from 1 mm to 1 m and frequencies between 300 MHz (λ = 1 m) and 300 GHz (λ = 1 mm) (Haque, 1999, Tang et al., 2010, Remya and Lin, 2011). The MW technology is widely applied in heating applications, and its unique operational principle offers many advantages over the conventional heating (Haque, 1999, Thostenson and Chou, 1999). For instance, in contrast to the conventional thermal processes the MW energy offers benefits such as high heating rates, interior heating, energy saving, greater control of the heating process, and higher level of safety and automation, among others (Haque, 1999, Thostenson and Chou, 1999). Heating of a material by MWs results from the rotation of dipolar species and/or polarization of ionic species due to their interaction with the electromagnetic field (Haque, 1999). The molecular rotation and migration of ionic species causes friction, collisions, and disruption of hydrogen bonds within water; all of which result in the generation of heat (Venkatesh and Raghavan, 2004). The ability of a material to absorb MW energy and subsequently get heated is governed by its dissipation factor, which is the ratio of the dielectric loss factor to the dielectric constant of the material. The dielectric loss factor depicts the amount of input MW energy that is lost by being converted (dissipated) to heat within the material while dielectric constant depicts the ability of material to delay or retard MW energy as it passes through. Hence, materials with high dielectric loss factors are easily heated by MW energy (Haque, 1999). A low loss material can be heated indirectly by MW energy by blending with a high loss material (i.e. MW facilitator e.g. char). The MW first heats the facilitator which then heats the low loss material by conduction (Haque, 1999, Menéndez et al., 2002).

FS, SS, and WAS contains high amount of dipolar molecules such as water and organic complexes, which makes them good candidates for the MW dielectric heating. Preliminary laboratory studies have demonstrated the efficiency of MW technology regarding sludge sanitization and volume reduction (Mawioo et al., 2016a, Mawioo et al., 2016b). For instance, bacterial removal below the detection limit was reported when sewage sludge (Hong et al., 2004, Hong et al., 2006) and blackwater sludge (Mawioo et al., 2016b) were exposed to MW irradiation. In addition, a reduction below the detection limit of both *E.coli* and helminth (*Ascaris lumbricoides*) eggs was achieved by exposing fresh FS to MW irradiation (Mawioo et al., 2016a). Furthermore, a volume reduction above 70% was attained by the MW treatment of anaerobic sewage sludge (Menéndez et al., 2002), blackwater sludge (Mawioo et al., 2016b), and fresh FS (Mawioo et al., 2016a). The mechanisms associated with the sludge treatment by MW regarding pathogen and volume reduction include thermal (temperature) and the non-thermal (electromagnetic radiation) effects of the electromagnetic energy (Banik et al., 2003, Hong et al., 2004, Mawioo et al., 2016b). A distinguished aspect of the MW treatment is a combined effect of thermal and non-thermal action involved in the destruction of microorganisms. The thermal effect causes rapturing of microbial cells when water is rapidly heated to the boiling point by rotating dipole molecules under an oscillating electromagnetic field (Tang et al., 2010, Tyagi and Lo, 2013). Conversely, the non-thermal effects cause disintegration by the breakage of hydrogen bonds, which is attributed to the rapidly changing dipole orientation in the polarized side chains of the cell membrane macromolecules (Banik et al., 2003, Park et al., 2004, Tyagi and Lo, 2013, Serrano et al., 2016). Thermal effects of the electromagnetic energy are linked to the sludge volume reduction (sludge dehydration) during the MW treatment. The resulting high temperature causes the vaporization and eventually removal of the water contained in the sludge (Mawioo et al., 2016b). Notwithstanding its success in achieving sludge sanitization and volume reduction, the MW treatment did not attain organic stabilization of sludge; presumably, due to the relatively low maximum temperature attained in the treatment process, i.e. 127 °C (Mawioo et al., 2016b) and 134 °C (Mawioo et al., 2016a). The need for high temperature for the effective removal of organic matter was demonstrated when sludge was mixed with a better MW receptor (facilitator) and then irradiated to attain temperature of over 900 °C (Menéndez et al., 2002).

These studies demonstrate the potential of application of the MW technology to treatment of various types of sludge. They were conducted at a laboratory scale using relatively small quantities of sludge, but the findings serve as a solid base for further scaling up of the MW-based sludge treatment technology. Furthermore, the research so far has been focused mainly on sludge sanitization and volume reduction and has not assessed the potential value for the valorization of the end-products. It is therefore desirable to evaluate the MW-based sludge treatment technology at a larger scale with larger sludge quantities, while simulating closely real field conditions. In addition, it is important to assess the resource potential of the process end-products (i.e. dry sludge in terms of its CV and nutrient content, and condensate/product water in terms of nutrient content and quantity). When successfully developed and tested, the MW technology can provide a viable alternative for dealing with the complex task of sludge treatment and disposal, especially in the conditions where from high amounts are generated such as the WWTPs and densely populated areas (e.g. slums and emergency settings), and other similar conditions (e.g. public events and religious gatherings).

The main objective of this study was to evaluate the MW efficiency (on the basis of selected parameters) on the treatment of several types of sludge including WAS from WWTPs, C-WAS, SS, and fresh FS. The research was conducted at pilot-scale, and the added value of the treatment end-products was evaluated. The study focused on four aspects, namely: reduction of selected pathogens, sludge volume reduction, organic matter reduction, and assessment of the value of treatment end-products. *E. coli*, coliforms, *Staphylococcus aureus* and *enterococcus faecalis* bacteria were used as pathogenic indicator microorganisms, while the sludge weight was used to estimate the volume reduction. CV, TN and TP content were used to assess the value of the treatment end products, while organic stabilization of the FS was estimated using the volatile to total solids ratio (VS/TS) comparison.

## 2 Materials and methods

### 2.1 Research design

This study was performed using four different sludges including WAS, C-WAS, SS, and FS. The differences of properties between the tested sludges allowed to assess the capability of the MW-based technology to treat each type of sludge with a variability that can be expected in real life full-scale field applications. The study was divided in four phases with each phase involving one type of sludge, starting with WAS and C-WAS (less demanding in terms of preliminary experimental handling). The next phase involved the treatment of SS, concluding with the treatment of fresh FS which was considered as the most challenging sludge sample to treat. Each time a 4 kg sludge sample was exposed to irradiation provided by MW generators (total installed power 3.4 kW) for a certain time period. Maximum contact time necessary to attain complete drying for each of the sludge types was pre-determined on the basis of preliminary trials. Parameters such as temperature, number of bacteria (i.e. *E. coli*, coliforms, *Staphylococcus aureus*, and *enterococcus faecalis*), weight/volume, and VS/TS ratio were identified to evaluate the MW unit's performance. These parameters were measured in both the raw (untreated) and treated sludge samples, and the differences were used to determine the reactor performance in time. The specific energy consumptions of the sludges at the various contact times were also calculated using the electrical energy supplied to the MW unit and the resultant loss in sludge weight/volume. Furthermore, to assess the value of the treatment end-products, additional parameters were measured for the treated sludge and the generated condensate at specific times during the experiment. These include COD, CV, TN, TP, and bacteriological indicators. All experiments were performed in triplicates. The experimental data was processed using Microsoft Excel software. In each case, the triplicate data for each set of contact time was combined by computing their mean values and the respective standard deviations and standard errors. The mean values were then presented in graphs with their respective standard error bars shown.

### 2.2 Experimental apparatus

**Fig. 1:**
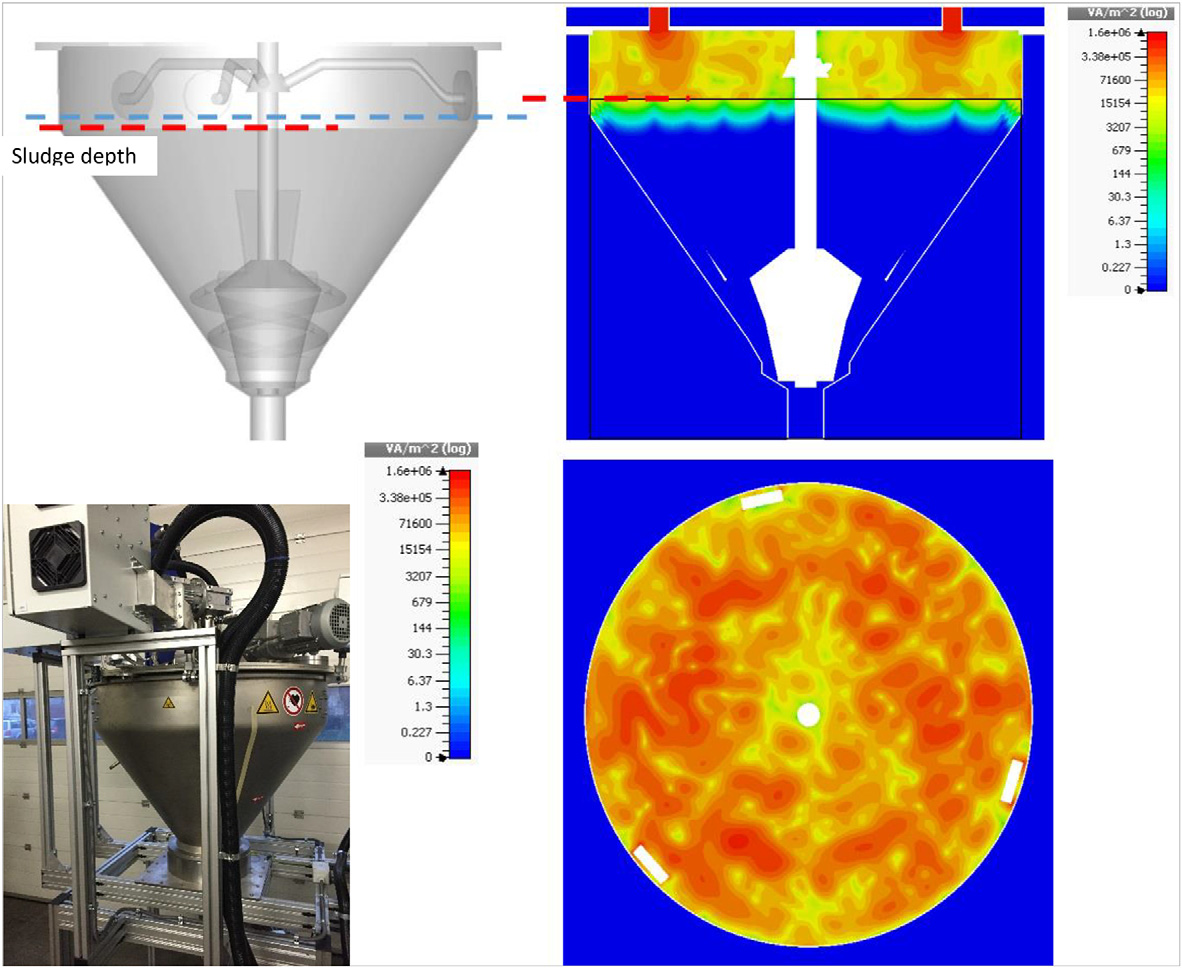
A MW model showing the power intensity distribution expressed as VA/m^2^ (W/m^2^) in the sludge during a MW treatment trial (courtesy of Fricke und Mallah Microwave Technology GmbH).

The pilot-scale MW reactor used in this study was designed by researchers at UNESCO-IHE Institute for Water Education (Delft, The Netherlands), and then manufactured in collaboration with Fricke und Mallah Microwave Technology GmbH (Peine, Germany) with additional modifications carried out by Tehnobiro d.o.o. (Maribor, Slovenia). A series of preliminary tests and modifications were carried out in the process of improving the prototype MW unit. The original design was based on a conical shape mixed reactor designed for the treatment of sludge in either continuous or batch mode. However, financial limitations allowed the purchase of 4 microwaves generators with a total installed power limited to 3.4 kW and an on-the-shelf cavity of a much larger volume than originally planned. The original evaluations on this first prototype with batches of 100 kg sludge confirmed that the principle works, but it took too long to heat up and dry the sludge in such a system. The results of a modelling study (shown in Fig. 1) were confirmed by practical observations that the MW irradiation was most efficient within the top part of the cavity (15–20 cm), in which the red color illustrates the area with the highest power intensity (hottest zones) and blue the area with the lowest power intensity (i.e. the coldest zones). This observation demonstrates that despite the MW capability to cause volumetric heating of material, there is an optimal depth by which the waves can penetrate a specific material. The penetration depths for specific materials should thus be considered when designing MW reactors.

**Fig. 2:**
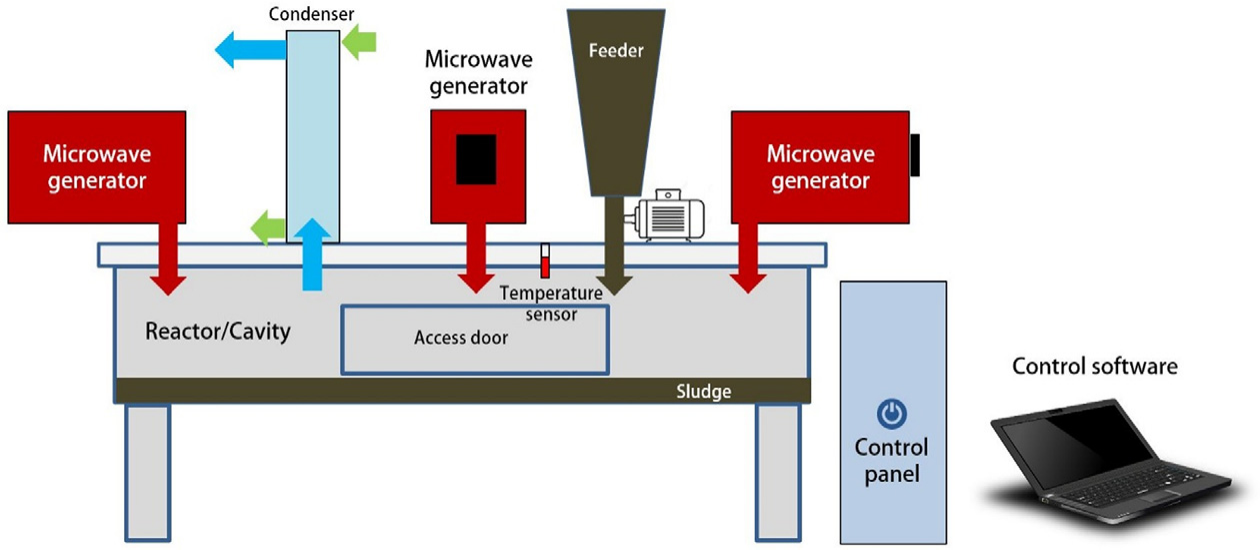
Schematic diagram of the microwave reactor unit.

A series of further improvements were carried out on the original design to achieve the final version of the reactor whose schematic diagram is shown Fig. 2.The improvements were done by introducing both a sludge recirculation pump, and an additional agitator to improve the sludge mixing and exposure to MW irradiation at the top of the reactor. The next step was to introduce a rotational tray at about 20 cm from the top of the cavity. In addition, the amount of sludge sample introduced to the reactor was reduced from 100 kg to approximately 10 kg. The bottom of the cavity was used as a storage of the sludge scraped off the tray still with the possibility to be recycled as long as the recycle pump could handle that sludge (version 2). Based on the experience with the first two versions of the prototype, it was decided to replace the conical cavity/reactor with a flat bottomed and relatively shallow unit without changing the arrangement on the top cover. Mixing and recycling was omitted in this unit, and the working capacity of the reactor was kept to approximately maximum of 30 kg sludge. However, it was decided to bring down the actual sample in the batch type of experimentation to 4 kg to better match the installed power capacity and decrease the exposure time. This third (final) version of the prototype was in fact used in this study (see Fig. 2).

The reactor unit (i.e. the final version) comprised a 196 L stainless steel cavity (i.e. the applicator) for holding the sludge during irradiation. The cavity was cylindrical in shape with a diameter of approximately 100 cm and a depth of 25 cm. Other features included four air-cooled MW generators, a condenser unit, an infrared temperature sensor, and a control unit. The four MW generators each with 850 W at 2450 MHz were installed on the top cover of the reactor. The condenser unit was also installed on the reactor cover for ease of vapor collection and odor control. The optris®CSmicro infrared temperature sensor (Optris GmbH, Germany) continuously measured the inside cavity temperature during the treatment. However, due to the instability noticed with the measurements by the sensor, a thermal camera (FLIR TG 165, FLIR Systems Inc., USA) was also used to measure the final (maximum) temperature of the irradiated sludge at the end of each treatment cycle. The temperature measured by the thermal camera was more reliable and thus was adopted for reporting in this study. Furthermore, the reactor was covered with a 10 cm fibreglass wool insulation layer with aluminium foil to reduce temperature losses to the surrounding environment. The ambient temperature (inside the industrial hall) was around 10 °C during the experimental period. As an extra measure of caution, a MW leak detector (Tenma 72–10,212, Tenma, China) was used to check any MW leakages around the reactor. All components of the control unit were integrated and supported by a control software (LabVIEW 8.0, National Instruments Corporation, USA), which was installed in a control computer (Dell laptop, Dell Inc., USA) that was used to control the operation and log the data. A power meter was used to measure the electrical energy supplied to the MW units, which together with the sludge weight/volume reduction was used to estimate the specific energy consumption at the various contact times.

The MW reactor was located at the workshop facility of Tehnobiro d.o.o. in Maribor, Slovenia where the entire experimental work was conducted during the period of 150 days (March to July 2016).

### 2.3 Sludge samples

The C-WAS, WAS and SS samples were collected from a municipal WWTP located in Ptuj, Slovenia. The WAS sample was collected from the WAS discharge line, while the C-WAS was obtained at the discharge point of the dehydrated WAS, which was thickened by the means of a centrifuge with the addition of a poly-electrolyte. The SS sample was collected from the trucks (while discharging at the WWTP) which collected the sludge from the septic tanks of the houses in the areas not covered by the sewerage. The fresh FS was obtained from urine diverting dry toilets (UDDTs) located at the Sustainability Park Istra (Gračišče, Slovenia). In each case, the samples were collected and transported in closed plastic containers to the research facility in the industrial zone of Maribor where they were stored at 4 °C prior to the experiments and analysis within 48 h.

### 2.4 Sample preparation and treatment

The sludge samples were treated using the MW apparatus. The samples were thoroughly mixed and weighted into batches of 4 kg and placed in plastic containers. They were then transferred into the MW reactor unit's cavity (i.e. applicator), evenly spread to attain a thickness of approximately 0.5 cm, and then exposed to the MW irradiation at 3.4 kW for variable time. Four batches each for the C-WAS and FS samples were separately exposed for the respective maximum periods of 30, 60, 90, and 120 min, while five batches each for the SS and WAS samples were separately exposed for the respective maximum periods of 30, 60, 90, 120, and 240 min. As a consequence of the MW treatment, vapor was produced from the heated sludge. The vapor was cooled down by a condenser and the generated condensate was collected in a plastic container separately for each test. After the MW treatment, the cavity door was opened and the sludge sample temperature was immediately measured. The irradiated samples were then cooled down and later analyzed for various parameters as described in the following section.

Various operational challenges were experienced at different stages of the treatment process. For instance, the loading process required spreading the sludge samples to achieve a relatively even thickness which consumed some time. This was particularly noticeable with the solid samples e.g. FS and C-WAS. It also took time to wait for the sludge temperature to cool down to a safe level for unloading, especially when samples were irradiated at longer contact time periods. Furthermore, dried samples got stuck on the cavity thus taking some time to scrape them off.

### 2.5 Analytical procedures

Several physical, chemical, and microbial parameters were measured in the sludge before and after the MW treatment and in the condensate. The sampling framework is shown in Table 1.

**Table 1:** Sampling framework.

Parameter/Media	WAS	C-WAS	SS	FS	Concentrate^a^
Temperature (°C)	●	●	●	●	
Weight-volume reduction ratio	●	●	●	●	
Total Solids (mg TS)	●	●	●	●	
Volatile Solids (mg VS)	●	●	●	●	
Calorific value (kJ/kg)	●	●	●	●	
Organic matter (mg COD/g TS)	●	●	●	●	●●
Total Nitrogen (mg N/g TS)^b^	●	●	●	●	●●
Total Phosphorus (mg P/g TS)^b^	●	●	●	●	●●
*E. coli* (CFU/g TS)	●	●	●	●	●●
Total coliforms (CFU/g TS)	●	●	●	●	●●
*Staphylococcus aureus* (CFU/g TS)	●	●	●	●	●●
*Enterococcus faecalis* (CFU/g TS)	●	●	●	●	●●

● Just before irradiation and immediately after the irradiation exposure.

●● At the end of the test, taken from the total collected condensate during the test.

a Condensate which is generated by treatment of each sludge sample.

b TN and TP concentrations were measured only after the MW treatment.

#### 2.5.1 Temperature measurement

The sample temperature was measured just before the exposure to microwaves. At the end of the each batch exposure, the cavity door was opened and the final (maximum) sample temperature was immediately measured in the reactor by a thermal camera (FLIR TG 165, FLIR Systems Inc., USA). The built in infrared temperature sensor (optris®CSmicro, Optris GmbH, Germany) located in the top plate of the cavity was continuously measuring the temperature, it was not accurate as it recorded the temperature of air close to the top of the reactor and not in the sludge sample.

#### 2.5.2 Weight/volume reduction calculation

The initial weight of the sample was determined using a weighing scale. After each treatment, the sample was cooled to room temperature and its weight was measured again. The volume reduction was then determined from the difference between the two measurements. Based on the maximum sludge temperature attained during MW treatment (i.e. ≤ 102 °C), the weight reduction could mainly be attributed to the water evaporated from the heated sludge. Thus, considering the density of water, the weight reduction was assumed to be equivalent to the sludge volume reduction.

#### 2.5.3 TS and VS measurements

TS and VS content of the sludge were determined according to the gravimetric methods (SM-2540D and SM-2540E, respectively, as described in APHA (2012).

#### 2.5.4 Calorific value measurement

The gross CV of the treated/dried sludge was measured in a bomb calorimeter (IKA- Kalorimeter C 400 adiabatisch IKA®-Werke GmbH & Co. KG, Staufen German) based on the ISO 1928:2009 Standard.

#### 2.5.5 Total COD measurement

Samples for COD measurement in the sludge and the condensate were prepared by diluting a known amount of sample in distilled water. The COD measurement was then conducted according to the open reflux method (SM 5220 B) (APHA, 2012). The values were expressed in mg COD per g TS (mg COD/g TS) or in mg COD per g liter (mg COD/L) for the condensate.

#### 2.5.6 TN and TP measurement

TN in the solid and liquid samples was measured using Dumas and Kjeldahl methods, respectively (Etheridge et al., 1998). TP in the solid and liquid samples were measured using the ascorbic acid method with acid persulfate digestion.

#### 2.5.7 Microbiological analyses

The detection of *E. coli*, coliforms, *Staphylococcus aureus*, and *enterococcus faecalis* was done using the surface plate technique. Chromocult coliform agar was used for both *E.coli* and coliforms (Chromocult; Merck, Darmstadt, Germany) (Byamukama et al., 2000). A step by step procedure as explained in Mawioo et al. (2016b) was applied to prepare and incubate the samples for the *E. coli* and coliforms analysis. Dark blue to violet colonies were classified as *E. coli* while red to pink colonies were identified as coliforms (Byamukama et al., 2000, Sangadkit et al., 2012). *Staphylococcus aureus* and *enterococcus faecalis* were grown using Baird-Parker RPF agar, and Slanetz and Bartley agar media, respectively (bioMérieux SA, Marcy l'Etoile, France). The preparation involved spreading 0.1 mL of the sample or its dilutions on the surface of the agar plate. The inoculated plates for *enterococcus faecalis* were incubated at 37 °C for 48 h and the colonies were identified as red, brown or pink. For the *Staphylococcus aureus*, the inoculated plates were incubated at 37 °C for 24 h but prolonged for an additional 24 h if no characteristic colonies were formed. Their colonies were identified as grey-black. The average number of colonies were used to calculate the viable-cell concentrations in the samples and expressed in either CFU/g TS or CFU/L of the test sample.

## 3 Results and discussion

### 3.1 Characteristics of the sludge samples

The sludge characteristics are presented in Table 2. FS exhibited the highest dry matter content followed by C-WAS, SS, and WAS, respectively. FS, C-WAS and WAS had a relatively high organic matter content (i.e. VS/TS over 0.70) compared to the SS with a VS/TS of 0.55. Furthermore, all measured pathogen indicators were detected in all sludges except the *E.coli* that was not detected in the C-WAS. As expected, the fresh FS appeared more polluted as it exhibited the highest pathogenic loads than all other sludges. The content of the various parameters as measured in the sludges were within the expected range. For instance, the values for TS and VS/TS in the FS were approximately 23% and 0.88, respectively, which is comparable to those reported in previous studies. Mawioo et al. (2016b) reported 26% and 0.92, while Rose et al. (2015) reported 25% and 0.84–0.93 for the TS and VS/TS ratio, respectively. Variations in the faecal composition can be expected due to diverse dietary intake of food and fluid (Rose et al., 2015). The TS (2%) and VS/TS ratio (0.76) values in the WAS were also comparable to those previously reported in other studies. For instance, Kim et al. (2003) reported a TS of 3.8% and VS/TS ratio of 0.68 for WAS while Tang et al. (2017) reported a TS of 2.1%. However, the TS value was relatively higher than the typical range (0.7–1.2%) (Takács and Ekama, 2008), which can be attributed to the fact that the sample was collected from the pipe delivering sludge to the centrifuges rather than in the clarifier. The TS content of the C-WAS (15%) was relatively lower than the typical 22–36% achieved using centrifuge, belt-filter press, and filter press dewatering (Metcalf and Eddy, 2003). Despite a high TS content (8%), SS had the lowest organic matter fraction with a VS/TS ratio of 0.55, which can be attributed to the high content of grit (observed) and the degradation of organic matter due to long retention time in the septic tanks.

**Table 2:** Characteristics of the raw sludge.

Parameter	Sludge type			
	C-WAS	FS	WAS	SS
Water content (%)	85	77	98	92
Total solids (TS, %)	15	23	2	8
VS/TS	0.76	0.88	0.76	0.55
TCOD (mg O_2_/g TS)	1.5	1.4	5.6	2.7
*E. coli* (CFU/g TS)	Not detected	4.23 × 10^8^	2.41 × 10^7^	1.03 × 10^5^
Coliforms	9.98 × 10^6^	3.58 × 10^7^	5.56 × 10^8^	3.42 × 10^6^
*Staphylococcus aureus*	3.00 × 10^7^	1.51 × 10^8^	9.74 × 10^7^	1.11 × 10^5^
*Enterococcus faecalis*	2.41 × 10^7^	4.55 × 10^8^	4.87 × 10^7^	1.46 × 10^5^

### 3.2 Temperature evolution during the tests

**Fig. 3:**
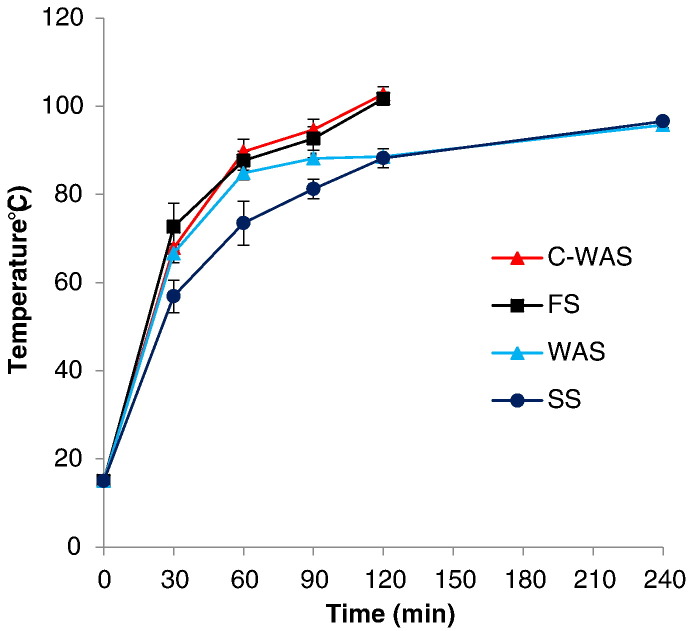
Temperature evolution in the sludge samples during the MW treatment.

Fig. 3 shows the temperature profiles for the respective sludge samples when exposed to MW irradiation. The temperature response of the samples to MW treatment appeared to vary among the sludge types. Generally, the temperature increment rate was relatively higher in the sample with low water content (i.e. C-WAS and FS), with both achieving a maximum of approximately 102 °C. Conversely, WAS and SS, which had a relatively high water content, exhibited a comparatively low increment rate to achieve a maximum temperature of approximately 96 °C. However, it can also be seen that C-WAS portrayed a higher rate of temperature evolution than the FS that had a lower water content. A similar trend in the temperature evolution rates was observed for the WAS in comparison to the SS.

Furthermore, as shown in Fig. 3, three distinctive temperature evolution phases were portrayed. These showed an initial rapid rise in sludge temperature that was followed by a fairly constant but minimal temperature rise, and finally again a fairly rapid temperature rise.

The variation in the temperature increment rates among the sludge samples evaluated can be attributed to their properties (Table 2), e.g. the water content, organic matter content, viscosity, etc. For instance, the samples with a higher water content (i.e. WAS and SS) demonstrated a relatively lower temperature increment rate. Water is a good dielectric material, but has a high thermal capacity, so more heat energy was initially consumed with a relatively smaller temperature increment in the samples with a higher water content compared to those with a lower water content (C-WAS and FS) (Mawioo et al., 2016b). This observation agrees with the previous studies in which different quantities and types of sludge with varying water contents were heated by microwaves (Tang et al., 2010, Mawioo et al., 2016a, Mawioo et al., 2016b). In addition, the different levels of organic matter among the sludges might have influenced their variation in temperature evolution. For example, WAS had a higher water content (98%) but still exhibited a higher temperature evolution rate than SS (92%), possibly due to its higher organic matter content i.e. VS/TS ratio of 0.76 compared to 0.55 in the SS. Carbohydrates, proteins and lipids, which are the primary organic components found in sludge (Tyagi and Lo, 2013) have high loss factors, hence their constituent amounts will definitely influence the sludge response to MW heating. Furthermore, the dry matter of the SS had a relatively high sand (quartz) content which has poor dielectric properties and is largely transparent to microwaves.

Conversely, the FS, which had a lower water but higher organic matter content than the C-WAS, exhibited a comparatively low temperature increase rate, which can be the effect of the viscosity. The FS sample was stickier, which can interfere with the movement of molecules and consequently the MW effect in the sludge. The MW irradiation effect is influenced by the friction of molecules in the medium, its change to heat energy, and the synergistic effect during irradiation through convection (Hong et al., 2004). Therefore, stickiness (viscosity) of a medium can influence temperature evolution during MW heating. However, the FS viscosity can be reduced by applying selected cover (odor control) materials such as char, etc., in the UDDTs so as to mix with FS in situ or by blending those materials with FS prior to the MW treatment. Besides odor control and viscosity improvement, char is a good MW receptor (has high loss factor) material that can enhance the MW irradiation process.

Despite attributing the differences in temperature evolution rates to the sludge properties, it was not possible to explicitly point out their specific range of influence. This can be achieved by conducting specific tests to determine the interactions of the various sludge properties with MW radiation, which was not in the scope of this study.

Furthermore, the temperature evolutions phases depicted in Fig. 3, are similar to observations reported in the previous studies, in which the three stages were classified in reference to the sludge drying phases, namely the preliminary, essential (major), and final drying phases (Flaga, 2005, Bennamoun et al., 2013, Bennamoun et al., 2014, Bennamoun et al., 2016, Mawioo et al., 2016b). Those phases were associated with the changes (e.g. heating and evaporation) occurring during the sludge drying period. For instance, the rapid temperature rise during the preliminary drying phase was linked to the initially high concentration of dipolar molecules (e.g. water, proteins, etc.) in the wet sludge that interacted with the microwaves to generate high amounts of heat (Yu et al., 2010, Chen et al., 2014, Mawioo et al., 2016b). On the other hand, the fairly constant but minimal temperature rise during the essential drying phase was associated with the fact that majority of the heat goes to the vaporization of the unbound water from the surface of the sludge particles while being constantly replaced from the inside (Flaga, 2005, Mawioo et al., 2016b). During the final phase, the rapid temperature rise was attributed to the more rapid evaporation of water from the surface of the sludge particles than it is replaced from the inside of the particle (Mawioo et al., 2016b).

It is also worth to mention that temperature measurements in this case was a challenge as it required first to switch off the MW generators (for operator safety) and then open the cavity door to facilitate the measurements using the thermal camera. Although this was done in a quick succession, the measured final (maximum) sludge temperature levels might probably be relatively lower than those actually attained as some heat loss could be expected, especially when the cavity door was opened. Such limitation can be addressed by installing an inbuilt temperature probe that is extended into the sample to replace the infrared sensor which was found to be unreliable as it recorded the temperature of air close to the top of the reactor and not in the sample.

### 3.3 Pathogen reduction

**Fig. 4:**
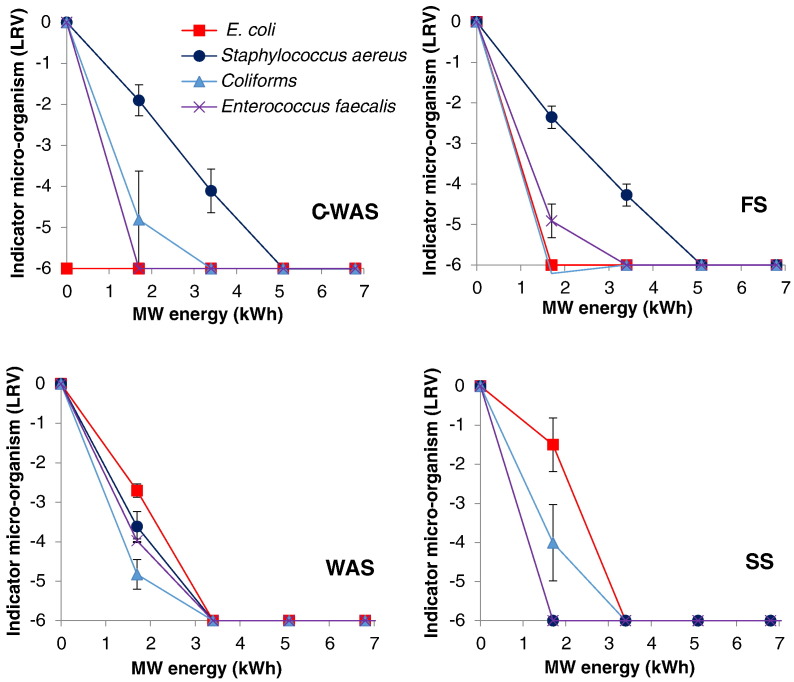
Effect of MW energy on reduction of indicator microorganisms in different sludges.

Fig. 4 shows the reduction profiles for the pathogens (selected indicator microorganisms) as function of the input MW energy for each type of sludge. The MW treatment was able to achieve complete destruction (below detection limit, i.e. < 500 CFU/g TS or approximately 5.90 log removal value (LRV)) of pathogens of interest in the sludge at the conditions evaluated in this work. In all samples, the destruction of pathogens increased with increasing MW energy. For instance, in all samples when the sludge was exposed at 1.7 kWh, an over 3 LRV was achieved for the coliforms and *enterococcus faecalis* bacteria. However, when the exposure to MW energy was raised to 3.4 kWh, a reduction beyond the detection limit was achieved.

Despite achieving a reduction below the detection limit for all selected pathogen indicator organisms in all the evaluated types of sludge, some disparities were observed concerning their die-off rate (Fig. 4). For instance, in the C-WAS and FS samples a relatively slower reduction was observed of *Staphylococcus aureus* (e.g. 2 LRV at 1.7 kWh) compared to both the coliforms and *enterococcus faecalis* (e.g. 4 LRV at 1.7 kWh). In addition, a reduction beyond the detection limit for the coliforms and *enterococcus faecalis* was observed at 3.4 kWh compared to the 5.1 kWh needed for *Staphylococcus aureus*. Furthermore, a higher reduction rate for the *Staphylococcus aureus* was observed in the samples with a higher initial water content (WAS and SS) compared to the samples with a lower initial water content (C-WAS and FS).

Moreover, the four pathogen indicator bacteria were measured in the product water/condensate, but none was detected.

The increment of the pathogen destruction with the increasing MW energy can be attributed to the resulting raise in both the thermal (temperature) effect and the electromagnetic radiation intensity (non-thermal effect), both of which are the mechanisms by which the microorganisms are destroyed during the MW treatment. Thermal effect is known to be the main mechanism by which the destruction occurs. Thermal effect causes rapturing of the microbial cells when water is rapidly heated to the boiling point by the rotating dipole molecules under an oscillating electromagnetic field (Tang et al., 2010, Tyagi and Lo, 2013). A temperature of approximately 70 °C has been reported as essential for the bacterial destruction (Hong et al., 2004, Valero et al., 2014). Microbial cells are also destructed by the non-thermal (electromagnetic radiation) effects by disruption of the hydrogen bonds that results when molecules or ionic species in the cell continually orient themselves in the direction of the changing electromagnetic field (Banik et al., 2003, Park et al., 2004, Tyagi and Lo, 2013, Serrano et al., 2016). Nevertheless, it is still difficult to distinguish between thermal and non-thermal effects in the relative contribution to pathogen kill.

The disparity concerning the rate of reduction, especially of the *Staphylococcus aureus* between the solids and the liquid samples can be linked to the sludge properties, e.g. dry matter content and viscosity, which might have affected the uniformity of the distribution of heat and electromagnetic radiation in the sample. A likely influence of the media properties was demonstrated in Walker and Harmon (1966) who observed varied thermal destruction rates of *Staphylococcus aureus* in skim milk, whole milk, cheddar cheese whey media, and a phosphate buffer.

Furthermore, the absence of the pathogen indicator bacteria in the product water/condensate can be attributed to the higher temperature necessary for water vaporization (i.e. 100 °C) compared to the essential 70 °C for bacteria destruction.

Generally, the results obtained here demonstrate that MW can offer an effective solution to sludge sanitization. The technology was capable to achieve complete reduction for all the pathogen indicator bacteria tested in the different sludge types. The results are in agreement with those obtained in the previous studies concerning pathogen destruction by MW treatment in which complete reduction of *E. coli,* coliforms, faecal coliforms, *Ascaris lumbricoides*, etc., was achieved (Border and Rice-Spearman, 1999, Hong et al., 2004, Lamb and Siores, 2010, Mawioo et al., 2016a, Mawioo et al., 2016b).

### 3.4 Weight or volume reduction and energy consumption

**Fig. 5:**
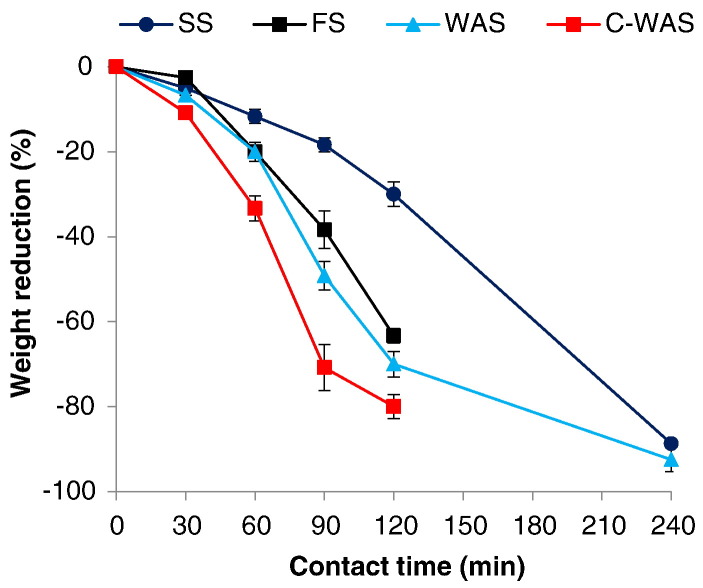
Effect of MW irradiation exposure time on the sludge weight.

The sludge weight/volume reduction results for the tested samples are shown in Fig. 5. The weight/volume reduction increased with the increasing exposure time, and the rate of reduction varied among the sludge sample types. The highest rate of reduction was observed in the C-WAS and the lowest in the SS. However, ultimately, the highest weight/volume reduction was realized in the SS followed by WAS, C-WAS, and FS in that order, with the respective reductions of approximately 93, 90, 80, and 63%. Furthermore, the three phases that were observed and described in the temperature evolution profiles, namely the preliminary, essential, and final drying phases were also reflected in the weight reduction profiles. In all cases, the preliminary drying phase occurred within the first 30 min of the processing time, and was marked by a minimal weight/volume reduction. A substantial weight reduction was realized in the subsequent essential (major) drying phase, in which over 60% weight reduction was achieved. Generally this phase lasted up to approximately 90, 120, and 240 min for the C-WAS, FS and WAS, and SS, respectively. The final drying phase, which is depicted by a decline of the weight reduction rate was only observed in the C-WAS between 90 and 120 min, and WAS between 120 and 240 min.

**Fig. 6:**
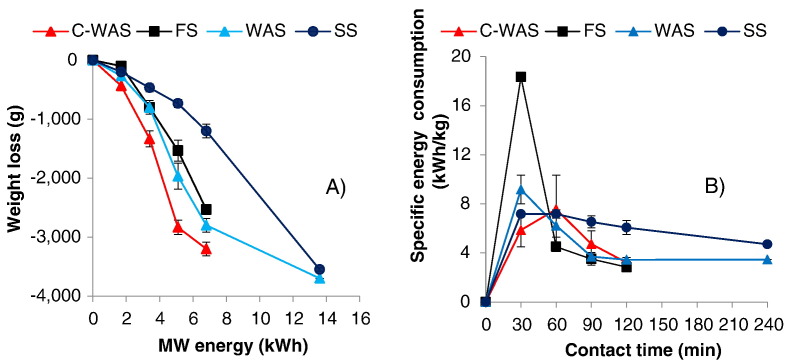
A) Sludge weight reduction and MW energy demand and, B) sludge specific energy demand as function of the MW exposure time.

In order to determine the energy demand during the MW treatment of the various sludges, the weight reduction along with the input MW energy profiles were observed (Fig. 6a). As can be seen, the trends correspond with the three drying phases that were observed in the temperature evolution and weight reduction. Furthermore, Fig. 6b, which presents the results for the specific energy consumptions (computed by use of the actual electrical energy consumed with the corresponding weight reductions) shows relatively high but varied energy demands among the sludge samples. In each case, the highest energy demand occurred during the preliminary drying phase. For instance, (and as shown in Fig. 6b) 18 kWh was required to achieve approximately 1 kg weight reduction in the FS, while 7–9 kWh was required to achieve approximately 1 kg reduction in the C-WAS, WAS, and SS during the preliminary phase. However, the energy consumptions at the maximum contact time were relatively lower at 3.2, 2.8, 3.5, and 4.6 kWh/kg for the C-WAS, FS, WAS, and SS, respectively. Nevertheless, they were higher than the theoretical energy demand i.e. 2.4, 2.1, 2.8, and 2.6 kWh/kg for the C-WAS, FS, WAS, and SS, respectively, that were estimated considering both the specific heat and the heat of vaporization of water.

The order in the weight/volume reduction trends as depicted in Fig. 5 was expected as those reductions result from vaporization of water, and are thus directly linked to the initial proportions of water and TS content in the sludge. Furthermore, the relationship between the three drying phases with the temperature profiles was anticipated since weight/volume reduction is linked to the evaporation of water, which is caused by the thermal (temperature) effects.

Generally, the results demonstrate that MW treatment was successful to achieve over 60% weight/volume reduction in all sludge types evaluated. Similar results have been achieved previously where even higher reductions over 70% were reported with the MW treatment (Menéndez et al., 2002, Mawioo et al., 2016a, Mawioo et al., 2016b). The disparity in the weight/volume reduction rates among the sludge samples was mainly attributed to their properties such as the initial water content, organic matter content, and viscosity, among others. As discussed in Section 3.2, these properties influence the temperature propagation and ultimately the moisture evaporation in the sample. Furthermore, the weight/volume reduction related drying phases (i.e. the preliminary, the essential and final drying phases) observed in this study agree with those reported in previous studies (Flaga, 2005, Mawioo et al., 2016a, Mawioo et al., 2016b). The minimum weight reduction in the preliminary phase was attributed to the high thermal capacity of water, which causes the absorption of a significant portion of the initially supplied MW energy, with a minimal temperature growth available for the evaporation of water (Mawioo et al., 2016a, Mawioo et al., 2016b). The essential drying phase, in which the majority of sludge weight/volume reduction occurs, is considered the most important stage in the MW treatment, especially if sludge weight/volume reduction is targeted. At this stage the supplied MW energy is largely used for the evaporation of the free (unbound) water that requires less energy (Mawioo et al., 2016b). As observed here with the C-WAS and FS, based on the initial thermal requirements, it is expected that the efficiency of volume reduction by MW treatment will be improved if the sludge has a higher TS content. Therefore, when possible, a pretreatment step (e.g. thickening or dewatering) can be introduced before sludge is brought to the MW treatment process. However, it would even be more prudent to promote the use of dry sanitation systems (e.g. UDDTs) in the areas that are feasibly targeted with the MW treatment (e.g. slums and emergency settlements).

Results from Fig. 6b show that specific energy consumption is the highest during the preliminary drying phase. This can be explained by the fact that the majority of the energy supplied during this phase is largely utilized to ramp the sludge and reactor temperature with very minimal sludge weight/volume reduction. The exceptionally high energy demand registered by FS during the preliminary phase can be attributed to its stickiness (high viscosity), which is among the factors that affect the capability of the electromagnetic (MW) radiation to penetrate a material (Hong et al., 2004). The general drop of the specific energy demand in the subsequent essential drying phase can be attributed to the fact that most of the resulting heat energy was directly used for weight reduction through the vaporization of water. Ultimately, the SS exhibited the highest energy demand (i.e. 4.6 kWh/kg), which can be attributed to the relatively high water content, low amount of organic matter, and high grit (poor MW absorber) content. Conversely, the relatively low energy demand exhibited by the FS (i.e. 2.8 kWh/kg) can be linked to its low water content (implying a reduced absolute thermal capacity and amount of moisture to evaporate) and the high organic matter content which promotes irradiation. It is expected that the specific demand for FS can be reduced further if the viscosity is reduced. Moreover, the specific energy demand variations among the tested samples demonstrated the influence of the irradiated material properties on the drying rate. For instance, C-WAS and WAS, being from a similar source, demonstrated very close results regarding the specific energy consumption.

Furthermore, from the results it appears that the amount of energy input to the system during the preliminary phase is important and can influence the overall performance. For instance, if the system's total installed power is relatively low, it would take longer to increase the sludge temperature to the boiling point. Consequently, this might promote any possible heat losses to the surrounding environment. Alternatively, a relatively high total installed MW power would fasten the heating process and likely reduce the overall energy demand. Temperature ramping can also be enhanced by blending sludge with a MW receptor material, e.g. char (Menéndez et al., 2002). This is particularly viable for FS generated from dry toilet facilities (e.g. UDDTs) where the char can be applied as cover material. Application of a cover material in the dry toilet facilities is usually recommended for odor control.

The specific energy consumptions obtained here are comparable with those obtained in previous studies (Mawioo et al., 2016a, Mawioo et al., 2016b), which reported 3 kWh per kg and 2.3–2.5 kWh per kg for fresh FS and blackwater sludge, respectively, after exposure to MW irradiation. However, results from those studies and the current study are still relatively higher than those reported for the convective and conductive industrial driers which vary between 0.7 and 1.4 and 0.8–1.0 kWh, respectively, per kg of vaporized water (Bennamoun et al., 2013). The disparities between the results of this study and those reported in Bennamoun et al. (2013) might be only to a lesser extend attributed to different properties of materials used in the study of Bennamoun and co-workers and four sludges used in this study. It is likely due to the fact that the novel MW-based technology developed in this study was not optimized regarding the energy consumption, which will be addressed in the next stage of development. Further improvements and optimization of the unit would definitely increase the performance and ultimately reduce the energy consumption. Enhancement of the condenser (vapor extraction) unit was identified as crucial to improve the efficiency of the moisture evaporation which ultimately might lower the unit's energy demand. Other factors that caused higher energy consumption in this study include the following: (i) each batch test was started with a cold reactor. A portion of the energy was initially used to warm up the reactor as well as sludge. The effect of the cold-start was not quantified in this study but can easily be checked in the future by comparison of the energy consumption between cold and hot start tests; (ii) the reactor insulation was sub-optimal. The reactor did not have sufficient thermal insulation, and this contributed to higher than desired energy consumption; and (iii) the tests were executed during the winter in cold environment (ambient temperature in the experimental hall was around 10 °C) and with cold sludge stored in the fridge. In addition, the space was very large and could not be heated up with the waste heat of the reactor. These are harsh conditions that are not representative for most of expected applications where ambient temperature is expected to be at least twice higher. These factors also contributed to the higher energy demands obtained in this study compared to the theoretical demands that were estimated considering only the specific heat and the heat of vaporization of water.

### 3.5 Energy and nutrients recovery

The MW treatment process converted the test sludge samples into three-phase products, namely solid (dried sludge), liquid (condensate), and gas. The results for the gross CV, TN, and TP measurements of the dried sludge are shown in Table 3. The dried FS exhibited the highest gross CV of 23 MJ/kg. C-WAS and WAS yielded 22 and 19 MJ/kg, respectively, while SS had the lowest CV of 16 MJ/kg. In addition, all the dried sludges contained expectedly a relatively high nutrient content, in which TN was generally higher than the TP across all the samples. C-WAS and WAS solids exhibited the highest TN concentration at 84 and 80 mg N/g TS, respectively; while FS and SS solids exhibited the lowest TN contents of 42 and 28 mg N/g TS, respectively. Similar trends were observed for the TP content. Furthermore, the TN and TP content were measured in the condensate and the results are presented Table 4. In a similar trend as the solids, the condensate demonstrated high concentration of TN but a relatively low concentration of the TP.

**Table 3:** Characteristics of the MW irradiated sludge at maximum exposure time applied.

Parameter	Sludge type			
	C-WAS	FS	WAS	SS
Water content (%)	5	8	3	2
Total solids (%)	95	92	97	98
VS/TS ratio	0.73	0.88	0.75	0.54
TN (mg/g TS)	84	42	80	28
TP (mg/g TS)	26	15	27	24
CV (MJ/kg)^a^	22	23	19	16
Specific energy consumption (MJ/kg)^b^	11.6	10.2	12.5	16.7

a Calorific value i.e. the energy contained in the dried sludge.

b The amount of energy (MJ) consumed to achieve a unit loss in sludge weight (kg).

**Table 4:** Nutrients content of the condensate from MW treated sludge.

Parameter	Sludge type			
	C-WAS	FS	WAS	SS
TN (mg/L)	2762	1377	49	213
TP (mg/L)	0.8	0.4	0.2	0.4
COD (mg/L)	385	76	15	34

The energy values can be associated with the VS content, which represents the combustible matter in the sludge. This is why FS, having the highest organic content, had the highest CV while SS had the lowest. In addition, the CV value for WAS obtained in this study (i.e. 19 MJ/kg) is comparable to the 18.75 MJ/kg that was reported in a previous study by Chen et al. (2014). The CVs obtained here were compared with those of the common biofuels. Both coffee husks and firewood have a gross CV of 16 MJ/kg (Diener et al., 2014) which was equivalent to the SS, the lowest among the evaluated sludge samples. The CVs for the sawdust and charcoal are 20 MJ/kg and 28 MJ/kg, respectively (Diener et al., 2014), and are comparable to the FS and C-WAS.

The high nutrient contents in the C-WAS and WAS is related to the activated sludge bacteria that sequesters those nutrients from the wastewater. The SS had a higher content of TP than the FS due to the fact that septic tanks receive mixed excreta and greywater that contains phosphorus from detergents while FS was sourced from a UDDT toilet excluding the urine, which normally contains the largest fraction of phosphorus released from the body (Rose et al., 2015). Moreover, the high TN content in the condensate derived from all sludges (as indicated in Table 4) can be attributed to the volatilization of ammonia (NH_3_) which is emitted from the hydrolysis of protein during sludge heating. Heating causes dissolution of the protein in sludge, then it hydrolyzes to form multipeptide, dipeptide and amino acid. The amino acid further hydrolyzes to form organic acid, NH_3_ and CO_2_ (Ren et al., 2006, Deng et al., 2009). Since NH_3_ is highly soluble in water, it is expected that the volatized fraction was completely dissolved during the condensation process. The nutrients content, especially the TN in both the solids and the condensate was relatively higher than in the bio-based fertilizers such as the compost manure. For instance, some studies have reported approximately 13 mg N/g TS in the compost manure (Agegnehu et al., 2016, Andreev et al., 2016).

Generally, the energy and nutrient results demonstrated a value addition of the end products (e.g. solids and condensate) from a MW based treatment process of the various sludges. The CVs of the dried sludges were relatively higher compared to the specific energy consumptions during the treatment in a rather energy non-optimized prototype. This suggests that a substantial amount of energy can still be recovered if the sludge is used as biofuel. However, the combustion byproducts of dried sludge and their eventual treatment need to be further assessed. An alternative way for resource recovery (e.g. nutrients) from the dried sludge is by applying it as a fertilizer or soil conditioner where applicable and allowed. Similarly, the condensate has high nutrients content and can be applied as fertilizer.

### 3.6 Organic matter reduction

**Fig. 7:**
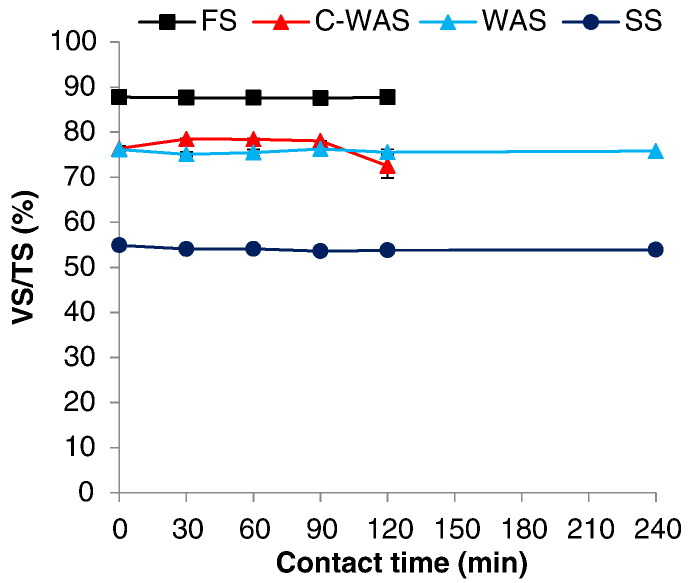
Effect of MW irradiation on the organic stability of sludge.

TS and VS fractions of the sludges were measured in the raw and treated samples and then used to compute the VS/TS values that are presented in Fig. 7. The VS/TS ratio was used as an index to determine the organic stability of the treated sludge (Bresters et al., 1997). The results show that there was no significant change in the VS/TS ratio between the raw and the MW treated sludge samples. The final VS/TS values were 76, 88, 73%, and 55% for the C-WAS, FS, WAS, and SS, respectively.

Based on the results, it can be deduced that the MW treatment in this case expectedly did not result in organic matter reduction. The final VS/TS, especially for the C-WAS, FS, and WAS were higher than the 0.60 recommended by European Environment Agency (Bresters et al., 1997) for the sludge organic stability. However, the VS/TS ratio for the untreated SS sample was lower than 0.60; hence, the sludge was already organically stable (which is expected for SS). The results obtained here agree with those reported in the previous studies in which different kinds of sludge were heated by MW. For instance, in a study by Mawioo et al. (2016a) final VS/TS of 0.86 and 0.92 were achieved for two sample fractions of MW treated FS. In yet another study, final VS/TS values of 0.80 and 0.89 were attained with the MW treatment of two sample fractions of blackwater FS, which were in the same range as the raw sludge (Mawioo et al., 2016b). The poor organic stabilization of sludge can be attributed to the comparatively low maximum temperatures attained by the MW treatment (i.e. 102 °C) than the 550 °C normally applied for VS ignition in the gravimetric method (SM-2540E) (APHA, 2012).

Although organic matter stabilization is not achieved, the sludge was made hygienically safe for further handling and the volume was significantly reduced. As demonstrated in Section 3.5, the dried sludge samples have relatively high (carbon) energy content and can be used as biofuel to promote resource recovery. Furthermore, volume reduction can minimize handling costs during disposal or further treatment (with less costly options like composting, etc.). The organic stability may not be a major consideration in situations of intensive toilet use, e.g. slums and emergency situations, provided that pathogens are inactivated and the public health risk is reduced.

### 3.7 Future outlook of MW application in sludge treatment

The results obtained in this study clearly demonstrate the suitability of the MW-based treatment of the various kinds of sludge from the perspective of both sanitization and volume reduction. The technology, when further developed and optimized particularly in terms of energy use, will be suitable for onsite applications and at the onset of the first phase of an emergency, when there are currently limited options available with the capacity to rapidly treat the large amounts of fresh sludge generated (Brdjanovic et al., 2015). In those and similar situations, pathogen inactivation should be prioritized over sludge volume reduction, as it is the most crucial aspect to curb possible excreta-related endemics. The choice for sanitization only will drastically shorten (by approximately 50–70%) the sludge exposure time and will strongly reduce the energy demand of the system. Furthermore, the technology showed high potential to complement waste activated sludge treatment at WWTPs as the biological sludge is comparatively less challenging to treat than fresh faecal sludge. It can also be considered as sole technology of (pre) treatment of septic sludge. Nevertheless, this being the early stages of the research and development of the prototype, it is clearly necessary to improve the various aspects of the technology, which will increase its efficiency and applicability at full scale. At the moment, the team at UNESCO-IHE is designing the next generation of a compact and energy efficient MW-based prototype that is integrated with liquid stream treatment and recovery of energy from dried sludge into a single containerized plant of capacity of 100 kg fresh faeces per hour. This mobile unit will be shortly tested in Slovenia and Croatia with several different sludges prior to its employment in Jordan where it will treat fresh faecal sludge from refugees in one of the camps.

## 4 Conclusions

A pilot scale MW-based prototype was developed, tested and evaluated on sanitization and volume reduction performances using four different kinds of sludge, namely partially dewatered/centrifuged waste activated sludge, fresh faecal sludge, septic tank sludge, and waste activated sludge. This study demonstrated that MW-based technology can be applied for rapid treatment of the different kinds of sludge, thereby providing a viable option for the treatment of sludge from intensively used toilet facilities (e.g. in slums and emergency settlements) and as complementary or sole treatment of waste activated sludge and/or septic tank sludge at WWTPs. A reduction below the detection limit was achieved of the pathogenic indicators. The technology resulted in a volume reduction of the raw material to over 60% and a high level of dry matter in the dried sludge was achieved (up to 98%). Besides, the process generated valuable end-products (condensate/product water and dry sludge) which can be recovered and reused as fuel, soil conditioner and/or fertilizer, etc.
